# Assessment of Fibrotic Liver Regeneration After Partial Hepatectomy With Intravoxel Incoherent Motion Diffusion-Weighted Imaging: An Experimental Study in a Rat Model With Carbon Tetrachloride Induced Liver Injury

**DOI:** 10.3389/fphys.2022.822763

**Published:** 2022-02-18

**Authors:** Shuangshuang Xie, Caixin Qiu, Yajie Sun, Yongquan Yu, Zhandong Hu, Kun Zhang, Lihua Chen, Yue Cheng, Mingzhu Bao, Quansheng Zhang, Jinxia Zhu, Robert Grimm, Wen Shen

**Affiliations:** ^1^Tianjin First Central Hospital, Tianjin Institute of Imaging Medicine, Tianjin, China; ^2^Siemens Healthcare (China), Beijing, China; ^3^Siemens Healthcare, Erlangen, Germany

**Keywords:** liver regeneration, liver fibrosis, partial hepatectomy, intravoxel incoherent motion imaging, liver function

## Abstract

**Purpose:**

To determine whether intravoxel incoherent motion (IVIM) parameters correlate with liver regeneration and function recovery after partial hepatectomy (PH) in rats with carbon tetrachloride (CCl_4_)-induced liver fibrosis.

**Methods:**

Sixty-two adult Sprague-Dawley rats were divided into the control group and the fibrosis group with CCl_4_ injection for 8 weeks. At the end of the 8th week, all rats received left lateral lobe liver resection. Within each group, IVIM imaging (*n* = 10/group) and histologic and biochemical analyses (*n* = 3/group/time point) were performed pre- and post-PH (on days 1, 2, 3, 5, 7, 14, and 21). Differences in liver IVIM parameters and correlation between IVIM parameters and Ki-67 indices, hepatocyte diameter, alanine transaminase (ALT), aspartate aminotransferase (AST), and total bilirubin (TBil) values were analyzed.

**Results:**

Post-PH, liver true diffusion coefficient (D) values decreased and pseudodiffusion coefficient (D*) and perfusion fraction (PF) values increased, then recovered to pre-PH levels gradually in both fibrosis and control rats. PF in fibrosis group were significantly higher than in controls from 3 to 21 days (*P* < 0.05). In fibrosis rats, both Ki-67 indices and hepatocyte diameters increased, and a strong correlation was found between PF and Ki-67 indices (*r* = −0.756; *P* = 0.03), D* and PF values and ALT, AST, and TBil values (*r* = −0.762 to −0.905; *P* < 0.05). In control rats, only hepatocyte diameters increased, and all IVIM parameters correlated well with hepatocyte diameters, ALT, AST and TBil values (*r* = 0.810 to −1.000; *P* < 0.05).

**Conclusion:**

The regeneration pattern in fibrotic liver tissue was different compared with control livers. IVIM parameters can monitor liver regeneration and functional recovery non-invasively after PH.

## Introduction

Liver regeneration (LR) is critical for the recovery of liver function after partial hepatectomy (PH). In most clinical scenarios, patients treated with PH have a history of chronic liver disease, and the abnormal, damaged liver is required to perform the regeneration. A previous study showed histologic evidence of liver regeneration in patients with cirrhosis or hepatitis (Nagasue et al., 1987). However, with liver fibrosis, total hepatocytes are decreased, which delays and impairs LR resulting in liver dysfunction (Ozaki, 2020). An accurate prediction of LR when the liver is compromised would enable clinicians to optimize PH approaches and improve liver surgery success rate.

In clinical medicine, liver volume measurements with computed tomography or magnetic resonance imaging (MRI) are commonly used as a measure of regeneration. But the relevance of this crude measure is not clear in the setting of more subtle interventions (Forbes and Newsome, 2016). Post-PH, the residual liver receives a blood supply that formerly perfused the entire liver. Hemodynamic regulation plays an important role in the initiation of LR (Yagi et al., 2020; Michalopoulos and Bhushan, 2021). Both hypertrophy and hepatocyte cell division contribute to LR (Miyaoka et al., 2012), which could affect the diffusion of water molecules. MRI offers a noninvasive option for assessing biomarkers that are associated with pathophysiologic changes. Additionally, intravoxel incoherent motion (IVIM) imaging provides perfusion and diffusion measurements of the liver (Le Bihan, 2008, 2019), which correspond to possible changes during LR. These measurements make IVIM imaging a possible alternative to liver biopsy for the assessment of liver fibrosis and inflammation (Tosun et al., 2020; Lefebvre et al., 2021), allowing the simultaneous evaluation of chronic liver disease severity and LR after PH.

In this study, we used IVIM to longitudinally observe liver changes in the control and carbon tetrachloride (CCl_4_)-induced fibrosis rat model of PH. The primary purpose was to investigate LR differences between control and fibrotic livers post-PH. Secondarily, our goal was to determine whether IVIM could be used as a noninvasive method to monitor the progress of LR and liver functional recovery post-PH.

## Materials and Methods

This study was approved by our Institutional Animal Care and Use Committee. All surgical procedures and MRI examinations were performed under inhalant anesthesia. First, rats were exposed to isoflurane (0.4 mmol/l; flow rate, 0.8 ml/s) in a chamber to induce anesthesia. Then, a close-fitting face mask covering the entire mouth and nose was used for continuous anesthesia during liver surgery or MRI examination. Isoflurane (0.15 mmol/l; flow rate, 0.5 ml/s) was supplied using flexible tubing connected to the mask.

### Study Design and Animals

A total of 62 adult male Sprague-Dawley rats (250–280 g) were used for experimentation. Liver fibrosis (*n* = 31) was induced by subcutaneous injection of CCl_4_ for 8 weeks. The CCl_4_ protocol included twice-weekly injections of a 40% (v/v) solution of CCl_4_ in olive oil. The first injected dose was 5 ml kg^–1^ body weight, followed by a dose of 3 ml kg^–1^ body weight. Weight-matched, untreated rats served as controls (*n* = 31) and did not undergo exposure to CCl_4_.

**FIGURE 1:**
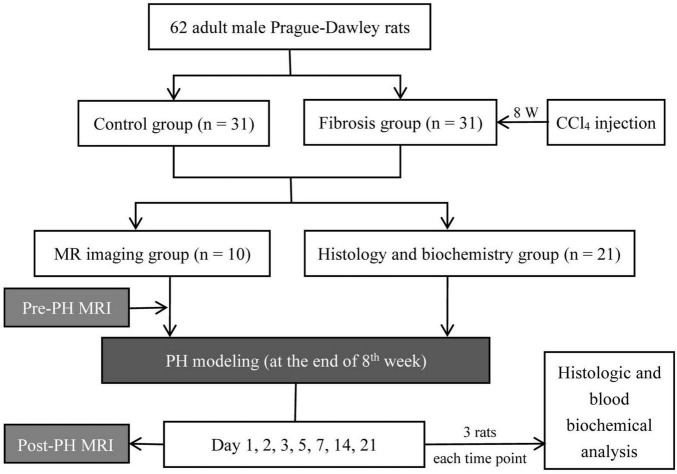
Flowchart of the experimental procedure; PH, partial hepatectomy.

In both the control and fibrosis groups, rats were divided into two subgroups, an MRI group (*n* = 10) and histology and biochemistry group (*n* = 21). In the MRI group, rats received a baseline MRI. Then, all rats received a left lateral lobe liver resection. Post-PH, MRI was performed on days 1, 2, 3, 5, 7, 14, and 21. Five rats (two in control group and three in fibrosis group) were excluded because they died due to anesthesia accident during the experimental period. In the histology and biochemistry group, histologic and blood biochemical analyses were performed at the same time points as for MRI. Three rats were used at each time point for a total of 21 animals. The portions of the liver resected during PH were used as samples for the baseline analysis of liver histology (Figure 1).

### Magnetic Resonance Imaging in Rats

All liver MRI examinations were performed with a 3T MRI scanner (MAGNETOM Prisma, Siemens Healthcare, Erlangen, Germany) with an eight-channel rat-specific coil (Chenguang, Shanghai, China). The rats were placed under anesthesia and restrained in the prone position to reduce respiratory motion. Axial T1-weighted imaging (T1WI) and T2WI were acquired for anatomic identifications, after which axial liver IVIM imaging was performed for quantitative parameter calculations. IVIM imaging, using a single-shot echo-planar imaging (EPI) sequence, was performed with the following parameters: repetition time (TR)/echo time (TE) = 2,300/74 ms; field of view (FOV) = 120 mm × 98 mm; slice thickness = 3 mm; matrix = 120 × 98; reconstructed voxel size = 0.5 mm × 0.5 mm × 3 mm; acceleration factor = 4 [generalized autocalibrating partially parallel acquisition (GRAPPA) = 2; slice acceleration factor = 2]; 10 *b* values (0, 10, 20, 30, 50, 75, 100, 300, 500, and 800 s/mm^2^) were applied in three diffusion directions, and acquisition time = 7 min 12 s.

### Image Analysis

Two expert radiologists with six (X.SS) and three (Q.CX) years of experience in abdominal image interpretation, respectively, and 2 years each of experience in rat liver MRI, analyzed the MR images independently. Post-processing of IVIM data and measurements were performed with an MR body diffusion toolbox (Siemens Healthcare, Erlangen, Germany). IVIM parameters, including the true diffusion coefficient (D), pseudodiffusion coefficient (D*), perfusion fraction (PF) maps, were extracted after fitting with a bi-exponential model. Parametric values were automatically output by measuring the region of interest (ROI). The largest three sections were chosen, and 2∼3 ROIs measuring 12∼16 mm^2^ were drawn on each section to measure the D, D*, and PF of the liver parenchyma, avoiding large vessels, artifacts, and liver borders. The average values of all ROIs were used for statistical analyses.

The two readers were blinded to the groups and imaging time points. Firstly, four rats were randomly selected (two rats in control group and two rats in fibrosis group). Their MR images at all time points were measured by the two readers independently to assess the inter-reader variability, and repeatedly by reader 1 after 1 month to assess the intra-reader variability. Then, all MR images of the remaining rats were measured by reader 1.

### Histologic and Biochemical Analyses

Liver samples were fixed in 4% phosphate-buffered formaldehyde and embedded in paraffin. Hematoxylin and eosin (HE) staining was performed for hepatocyte size, Masson staining was performed for fibrosis stage, and immunostaining was performed for Ki-67 (1:100; Abcam, United Kingdom). All pathologic specimens were reviewed by a pathologist with 10 years of experience in liver pathology (H.ZD). The diameter of hepatocytes were determined by measuring 10 hepatocytes in high-power fields (×400 magnification), and the values were averaged. The degree of liver fibrosis was graded using the Ishak score (S0∼S6) (Zhao et al., 2008). The ratio of Ki-67 positive-staining hepatocytes was determined by manual counting in 5 random high-power fields (×400 magnification).

Four ml venous blood was extracted though the marginal ear vein and centrifuged for 6 min at a speed of 3,000 r/min. Serum levels of alanine transaminase (ALT), aspartate aminotransferase (AST), and total bilirubin (TBil) were measured to evaluate liver function.

### Statistical Analysis

Continuous variables are expressed as the mean ± standard deviation, and categorical variables are expressed as a frequency or percentage. The Shapiro–Wilk test was used to evaluate data distributions. Repeated-measures ANOVA was used to compare IVIM parameters in each group at different time points. The multivariate analysis of variance was used to compare IVIM parameters between the two groups at each time point. The correlation between IVIM parameters and histologic and blood biochemical indices were analyzed by Spearman analysis. The significance threshold was set at *P* < 0.05. Inter- and intra- observer reliability for measuring IVIM parameters was determined by calculating the intra-class correlation coefficient (ICC) and Bland-Altman analysis. The agreement of measurements was considered poor (ICC < 0.50), moderate (0.50 ≤ ICC < 0.75), good (0.75 ≤ ICC < 0.90), or excellent (ICC ≥ 0.90) (Koo and Li, 2016). All statistical analyses were performed using SPSS 22.0.

## Results

### Intravoxel Incoherent Motion Parameter Analyses

**FIGURE 2:**
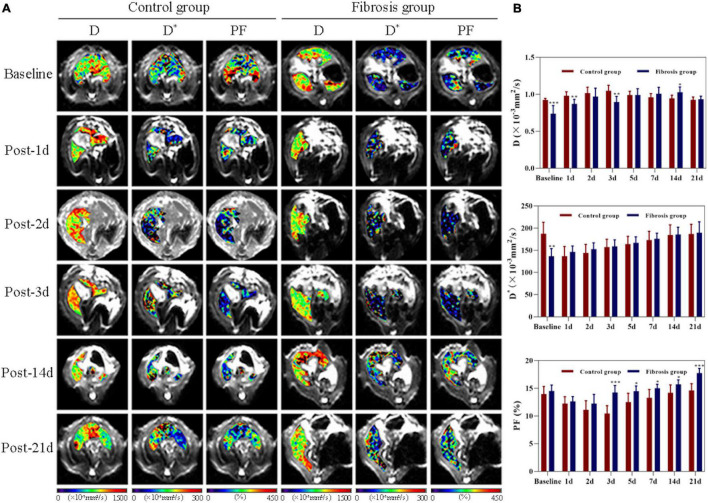
Liver changes showed in D, D*, and PF maps derived from IVIM imaging. **(A)** Changes in control and fibrosis groups on baseline and day 1, 2, 3, 14, and 21 post-PH. **(B)** Changes and comparison of liver D, D*, and PF values between control and fibrosis groups are shown at all time points before and after surgery. IVIM, intravoxel incoherent motion; PF, perfusion fraction; PH, partial hepatectomy. *P < 0.05; **P ≤ 0.01; ***P ≤ 0.001.

There were significant differences in liver D, D*, and PF values among all time points in both control and fibrosis groups (*P* < 0.001, Figure 2). Pre-PH, liver D, and D* values in the fibrosis group were significantly lower compared with the control group (D: −0.18; 95% CI, −0.3 to −0.1; *P* < 0.001; D*: −50.73; 95% CI, −75.5 to −25.9; *P* = 0.001).

Liver D values increased significantly on day 1 post-PH in both groups (*P* < 0.05). On day 21 post-PH, liver D values returned to baseline levels in the control group (0.003; 95% CI, −0.03 to 0.04, *P* = 0.828), but remained elevated above baseline in the fibrosis group (0.195; 95% CI, −0.07 to 0.31; *P* = 0.008), and there was no statistical difference in liver D values between the control and fibrosis groups (−0.009; 95% CI, −0.05 to 0.04; *P* = 0.679).

Liver D* values decreased significantly on day 1 post-PH in controls (*P* < 0.05) and returned to baseline levels by day 14 post-PH (−3.07; 95% CI, −12.6 to 6.5; *P* = 0.471). However, liver D* values increased significantly on day 1 post-PH in the fibrosis group (9.58; 95% CI, 2.7 to 16.4; *P* = 0.014), and continued to gradually increase at all post-PH time points (*P* < 0.05). There was no statistical difference in the liver D* values between the control and fibrosis groups at any post-PH time points (*P* > 0.05).

Liver PF values decreased significantly on day 1 post-PH in both groups (*P* < 0.001), and reached a minimum on day 3 post-PH in the control group (−3.52; 95% CI, −4.0 to −3.1; *P* < 0.001) and on day 2 post-PH in the fibrosis group (−2.28; 95% CI, −3.4 to −1.2; *P* = 0.002). Liver PF values returned to baseline levels by day 5 post-PH in the control group (−1.43; 95% CI, −3.1 to 0.2; *P* = 0.11) and day 3 post-PH in the fibrosis group (−0.25; 95% CI, −0.9 to 0.4; *P* = 0.38). However, PF values rose significantly above baseline in the fibrosis group by day 14 post-PH (1.22; 95% CI, 0.2 to 2.2; *P* = 0.026), and increased further by day 21 post-PH (3.23, 95% CI, 2.0 to 4.4; *P* = 0.001). Liver PF values in the fibrosis and control groups were similar pre-PH and on days 1 and 2 post-PH (*P* > 0.05). The PF values were significantly higher in the fibrosis group than the control group from days 3 to 21 post-PH (*P* < 0.05) (Table 1).

**TABLE 1:** Comparison of IVIM parameters in control and fibrotic liver tissue groups after left lateral lobe resection.

	D ( × 10^–3^mm/s)			D* (×10^–3^mm^2^/s)			PF (%)		
Time	Control group	Fibrosis group	*P-*value	Control group	Fibrosis group	*P-*value	Control group	Fibrosis group	*P-*value
Baseline	0.9 ± 0.1	0.7 ± 0.1	<0.001^#^	187.5 ± 25.8	136.8 ± 17.0	0.001^#^	14.0 ± 1.4	14.5 ± 1.1	0.401
1 day	1.0 ± 0.1*	0.9 ± 0.1*	0.002^#^	136.8 ± 21.8*	146.4 ± 13.2*	0.330	12.3 ± 1.2*	12.7 ± 0.9*	0.483
2 days	1.0 ± 0.1*	1.0 ± 0.1*	0.376	143.8 ± 19.6*	152.5 ± 14.4*	0.350	11.1 ± 1.7*	12.2 ± 1.7*	0.212
3 days	1.1 ± 0.1	0.9 ± 0.1*	0.002^#^	157.4 ± 17.6*	159.1 ± 14.4*	0.844	10.4 ± 1.4*	14.3 ± 1.2	<0.001^#^
5 days	1.0 ± 0.1*	1.0 ± 0.1*	0.950	164.0 ± 15.5*	167.0 ± 13.4*	0.720	12.5 ± 1.6	14.5 ± 1.0	0.013^#^
7 days	1.0 ± 0.1*	1.0 ± 0.1*	0.201	173.3 ± 19.6	176.1 ± 12.6*	0.747	13.3 ± 1.5	15.0 ± 0.7	0.015^#^
14 days	1.0 ± 0.0*	1.0 ± 0.1*	0.016^#^	184.5 ± 23.0	185.8 ± 16.2*	0.898	14.2 ± 1.4	15.7 ± 0.8*	0.024^#^
21 days	0.9 ± 0.0	0.9 ± 0.0*	0.679	187.0 ± 21.7	190.1 ± 24.1*	0.796	14.6 ± 1.2	17.7 ± 0.9*	<0.001^#^
*F*-value	13.098	13.716		58.505	83.643		80.340	53.713	
*P*-value	<0.001^†^	<0.001^†^		<0.001^†^	<0.001^†^		<0.001^†^	<0.001^†^	

*Unless otherwise indicated, data are mean ± standard deviation.*

*^†^< 0.05 versus all time points.*

*< 0.05 versus Pre.

*^#^< 0.05 versus two groups.*

*IVIM, intravoxel incoherent motion.*

ICC and Bland-Altman analysis showed good to excellent inter- and intra-reader agreement for measurements of liver D, D* and PF values (D: ICC, 0.861−0.951; D*: ICC, 0.842−0.984; PF: ICC, 0.886−0.984) (Table 1 and Supplementary Figure 1).

### Histologic and Blood Biochemical Analyses

#### Ki-67 Indices

**FIGURE 3:**
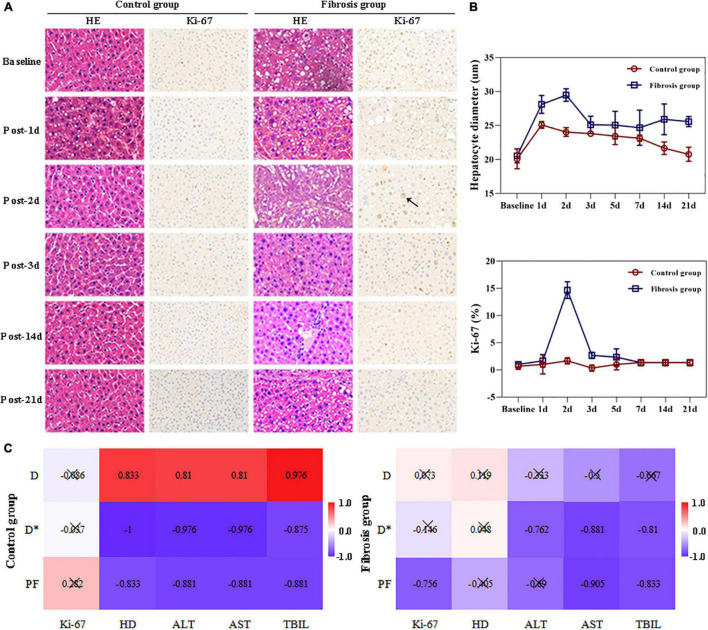
Liver histopathologic changes and correlation analysis. **(A)** Hematoxylin and eosin stain (HE) showed enlarged hepatocytes post-PH in both the control and fibrosis groups. Immunohistochemical staining of Ki-67 (brown) showed increased Ki-67^+^ cells (black arrow) in the fibrosis group 2 days after surgery. **(B)** Changes in hepatocyte diameters and Ki-67 indices post-PH. **(C)** Correlation hot maps between liver IVIM parameters and LR-related histologic results and liver function biochemical indices. ALT, alanine transaminase; AST, aspartate aminotransferase; HD, hepatocyte diameter; LR, liver regeneration; PH, partial hepatectomy; TBil, total bilirubin.

Hepatocyte Ki-67 indices in the control group showed no obvious changes post-PH. In the fibrosis group, hepatocyte Ki-67 indices were similar to those of the control group pre-PH, and were significantly increased on day 2 post-PH, decreased on day 3 post-PH, and returned to baseline levels by day 7 post-PH (Figures 3A,B).

#### Hepatocyte Diameters

Hepatocyte diameters were similar between the two groups at baseline and increased on day 1 post PH in both groups. The diameters returned to baseline levels by day 14 in the control group. In the fibrosis group, hepatocyte diameters increased to maximum levels on day 2, then decreased slightly on day 3, but remained elevated above baseline from days 3 to 21. Hepatocyte diameters in the fibrosis group were significantly greater than that of controls at all time points post-PH (Figures 3A,B).

#### Fibrosis Grades

**FIGURE 4:**
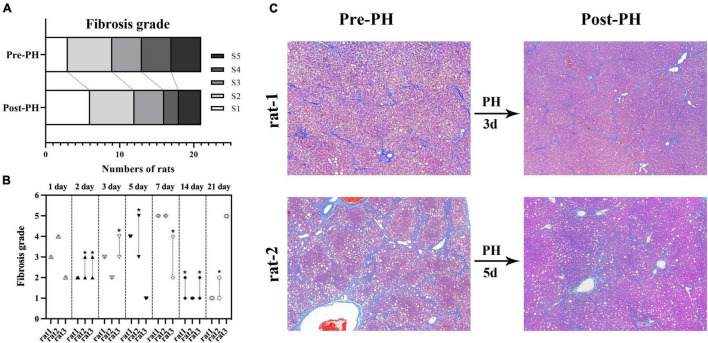
Fibrosis grade reversal after PH in fibrosis group **(A)**. Changes of fibrosis grades of all rats in fibrosis group. Eight rats had a decreased fibrosis grade (*, the upper and lower symbols correspond to fibrosis grade before and after PH, respectively), and the other 21 rats showed no change **(B)**. A representative example in rat-1, Masson’s trichrome (MT) staining of all liver samples from baseline and 3 days post-PH showed grade 3 liver fibrosis. A representative example in rat-2, MT staining of liver samples from baseline showed grade 5 liver fibrosis, and the sample from 5 days post-PH shows liver grade 3 liver fibrosis **(C)**. CCl_4_, carbon tetrachloride; PH, partial hepatectomy.

Resected liver samples from rats in the histologic and biochemical groups (*n* = 21) had grades of S1, S2, S3, S4, and S5 in 3, 6, 4, 4, and 4 rats, respectively. Post-PH, liver samples from all analytic time points had grades of S1, S2, S3, S4, and S5 in 6, 6, 4, 2, and 3 rats, respectively. The fibrosis grade decreased in 38% (8/21) of the rats, and no change was seen in the other 62% (13/21) (Figure 4).

#### Blood Biochemical Indices

Pre-PH, ALT, AST, and TBil levels in the fibrosis group were significantly higher than in the control group. Post-PH, a significant increase in ALT (approximately 1.5–2.5 fold), AST (approximately 3–4 fold), and TBil levels (approximately 3–5 fold) were seen on day 1 in both groups. By day 5 post-PH, ALT, and AST decreased back to baseline levels. TBil levels increased significantly on day 2, followed by a significant decrease on day 3 post-PH. In the fibrosis group, TBil levels were significantly lower from days 3 to 21 post-PH compared with pre-PH levels and were similar to controls (Table 2).

**TABLE 2:** Changes in laboratory and histologic findings in control and fibrotic liver tissue groups after left lateral lobe resection.

	ALT (U/L)		AST (U/L)		TBil (μmol/L)		Ki-67 index (%)		HD (μm)	
	Control group	Fibrosis group	Control group	Fibrosis group	Control group	Fibrosis group	Control group	Fibrosis group	Control group	Fibrosis group
Baseline	50.7 ± 11.9	201.0 ± 30.8	84.8 ± 10.2	527.7 ± 139.9	1.05 ± 0.2	11.3 ± 4.0	0.7 ± 0.6	1.0 ± 0.0	20.1 ± 1.4	20.5 ± 1.0
1 day	84.8 ± 10.2	564.1 ± 77.1	316.0 ± 136.2	1620.0 ± 149.0	5.01 ± 0.7	32.6 ± 4.1	1.0 ± 1.7	1.7 ± 1.2	25.1 ± 2.7	28.1 ± 1.3
2 days	99.4 ± 42.9	524.7 ± 209.0	151.6 ± 37.9	1828.6 ± 668.2	39.8 ± 16.1	39.4 ± 13.0	1.7 ± 0.6	14.7 ± 1.5	24.0 ± 3.0	29.5 ± 0.9
3 days	60.9 ± 18.0	64.0 ± 15.1	128.1 ± 31.0	115.2 ± 9.4	5.5 ± 1.0	6.4 ± 4.5	0.3 ± 0.6	2.7 ± 0.6	23.8 ± 2.8	25.1 ± 1.3
5 days	55.6 ± 9.2	66.6 ± 5.4	102.0 ± 7.7	99.3 ± 18.0	5.1 ± 2.6	3.3 ± 0.3	1.0 ± 1.0	2.3 ± 1.5	23.5 ± 3.9	25.1 ± 2.0
7 days	52.8 ± 9.3	63.4 ± 15.1	99.8 ± 12.9	81.3 ± 8.0	3.7 ± 2.7	2.1 ± 1.1	1.3 ± 0.6	1.3 ± 1.5	23.1 ± 2.0	24.7 ± 2.6
14 days	52.0 ± 6.3	66.6 ± 5.4	88.3 ± 11.4	87.2 ± 10.0	3.2 ± 1.3	2.2 ± 0.2	1.3 ± 0.6	1.3 ± 0.6	21.7 ± 2.5	25.9 ± 2.3
21 days	47.7 ± 6.1	57.8 ± 5.9	83.3 ± 7.8	78.1 ± 5.5	2.0 ± 0.8	2.3 ± 1.1	1.3 ± 0.6	1.3 ± 0.6	20.8 ± 3.3	25.6 ± 0.7

*ALT, alanine transaminase; AST, aspartate aminotransferase; HD, hepatocyte diameter; TBil, total bilirubin.*

#### Correlation Analyses

In the fibrosis group, liver PF values were highly correlated with hepatocyte Ki-67 indices, AST, and TBil levels (*r* = −0.756, −0.905, −0.833, respectively; *P* ≤ 0.05). Liver D* values were highly correlated with ALT, AST, and TBil levels (*r* = −0.762, −0.881, −0.810, respectively; *P* < 0.05).

In the control group, all liver D, D*, and PF values were highly correlated with hepatocyte diameter (*r* = 0.833, −1.000, −0.833, respectively; *P* < 0.05), ALT (*r* = 0.810, −0.976, −0.881, respectively; *P* < 0.01), AST (*r* = 0.810, −0.976, −0.881, respectively; *P* < 0.01), and TBil levels (*r* = 0.976, −0.857, −0.881, respectively; *P* < 0.01; Figure 3C and Table 3).

**TABLE 3:** Correlation matrix for IVIM parameters and laboratory and histologic measurements in the control and fibrosis liver tissue groups.

	D (×10^–3^mm^2^/s)		D* (×10^–3^mm^2^/s)		PF (%)	
Parameter	*r-*value	*P-*value	*r-*value	*P-*value	*r-*value	*P-*value
**Fibrosis group**						
ALT (U/L)	−0.333	0.420	−0.762	0.028*	−0.690	0.058
AST (U/L)	−0.500	0.207	−0881	0.004*	−0.905	0.002*
TBil (μmol/L)	−0.667	0.071	−0.810	0.015*	−0.833	0.010*
Ki-67 index (%)	0.073	0.863	−0.146	0.729	−0.756	0.030*
HD (μm)	0.119	0.779	0.048	0.911	−0.405	0.320
**Control group**						
ALT (U/L)	0.810	0.015*	−0.976	<0.001*	−0.881	0.004*
AST (U/L)	0.810	0.015*	−0.976	<0.001*	−0.881	0.004*
TBil (μmol/L)	0.976	<0.001*	−0.857	0.007*	−0.881	0.004*
Ki-67 index (%)	−0.086	0.840	−0.037	0.931	0.282	0.498
HD (μm)	0.833	0.010*	−1.000	<0.001*	−0.833	0.010*

ALT, alanine transaminase; AST, aspartate aminotransferase; IVIM, intravoxel incoherent motion; TBil, total bilirubin; HD, hepatocyte diameter. *P < 0.05.

## Discussion

In this study, LR post-left lateral lobe resection was evidenced by increasing hepatocyte sizes with cell division in the rats with fibrosis, whereas increasing hepatocyte sizes were observed without increased cell division in the control rats. This demonstrates different regenerative modes between normal and fibrotic liver tissue. In addition, liver IVIM parameters showed significant changes after PH and strong correlations between IVIM parameters and LR and liver functional indices, indicating the possible use of these parameters as noninvasive biomarkers to monitor LR and functional liver recovery post-PH.

Previously, Eberhardt et al. (2018) reported that apparent diffusion coefficients of liver tissues derived from conventional diffusion-weighted imaging decreased after major PH, then converged toward baseline values. Sheng et al. (2018a,b) reported that liver D values derived from diffusion kurtosis imaging could provide added value in evaluating the microstructures of liver regeneration in portal vein ligation for a staged hepatectomy model. However, tumors occurring in the fibrotic liver tissue were very common in clinical practice, and the authors only investigated liver diffusion changes after PH in normal liver. Our study established a PH model in normal and fibrotic livers. First, the results show a significant decrease in liver D values in fibrotic livers compared with control livers. This was consistent with previous animal and clinical studies (Chow et al., 2012; Tosun et al., 2020), demonstrating the significance of D values measured with IVIM imaging in detecting liver fibrosis. Second, results clearly show increased liver D values derived from IVIM imaging in both control and fibrotic livers on day 1 after left lateral lobe resection, followed by convergence toward baseline levels in the control livers; however, the D values continued to be increased over baseline levels in the fibrotic livers on day 21. These changes in liver D values after left lateral lobe resection were contrary to the previous portal vein ligation model that could have been due to the use of a different LR model and a different proportion of damaged liver. Further studies on varying PH amounts are necessary. In our study, liver D values showed a strong correlation with hepatocyte diameters in the control livers, indicating the potential value of D values in reflecting LR processes.

Post-PH, residual liver receives a large blood supply that can cause abrupt and drastic hemodynamic changes. Harkness (1952) found that residual liver was much paler than control livers up to 48 h post-PH, and liver blood space decreased to a minimum at 48 h, followed by a gradual increase after a 67% hepatectomy in control livers. Jin et al. (2018) found liver blood flow increased on day 1 and then decreased on day 5 after 45% PH by laser speckle contrast analysis in cirrhotic livers. However, those methods were invasive. In our study, liver D* and PF values derived from IVIM imaging were used to non-invasively measure liver perfusion changes after PH. Pre-PH, the results showed a significant decrease in liver D* values and no obvious change in PF values in fibrotic livers compared to control livers. These findings were consistent with a previous animal study (Chow et al., 2012), the decrease of D* values resulted from the alteration in liver perfusion during the progression of liver fibrosis. Post-PH, the results clearly showed decreased liver D* values on day 1 and then gradual recovery in controls, but a continuous increase of liver D* values in the fibrosis group, findings consistent with previous studies (Harkness, 1952; Jin et al., 2018). The opposing changes in liver D* values between control and fibrotic livers in the early stage post-PH might result from the different microstructure and response of residual liver to PH. From day 2 post-PH, the trend and magnitude of increasing liver D* values were consistent between the control and fibrosis groups. In our study, liver PF values decreased to a minimum on day 3 in controls and day 2 in the fibrosis group post-PH, and liver PF values in the fibrotic livers were higher than in the control livers starting at day 3 post-PH. In control rats, the time of liver PF values required to decrease to minimum was consistent with that of liver sinusoidal density (Nagasue et al., 1987). In fibrosis rats, our histologic analysis showed an increase in hepatocyte Ki-67 indices, which did not occur in controls, and liver PF values correlated well with hepatocyte Ki-67 indices. Liver regeneration requires a rich supply of oxygen and energy (Alexandrino et al., 2017). Higher liver PF values in the fibrotic livers post-PH could have been due to the increased blood supply from the hepatic artery. Liver perfusion quantification from IVIM was unable to separate portal veins from hepatic artery blood delivery. Further studies on perfusion changes from the hepatic artery post-PH are necessary. In addition, we evaluated the changes of fibrosis grades post-PH and found that 38% (8/21) of rats had a decreased fibrosis grade, and the other 62% (13/21) showed no change. This indicated the potential of LR after PH to reverse liver fibrosis. Liver D* values decreased in severe fibrosis (Luciani et al., 2008), but increased in early fibrosis (Tosun et al., 2020). So, our high liver D* values post-PH compared with baseline in the fibrotic livers might have been partially due to the reversal of liver fibrosis during PH.

Liver function analysis showed ALT, AST, and TBil levels were higher in the fibrosis group compared to controls at baseline. Post-PH, all blood biochemical indices increased significantly, especially in the fibrosis group, then decreased gradually. This trend of change was consistent with a previous study (Jin et al., 2018). However, all indices decreased significantly on day 3 post-PH in our study but remained high in a previous report (Jin et al., 2018). A possible explanation for this discrepancy might be due to the different levels of liver resection and injection times of CCl_4_ (8 weeks in our study vs. 10 weeks in the previous study). In addition, blood biochemical indices decreased below baseline levels in the fibrosis group starting at day 3 post-PH, which indicates liver injury recovery. The correlation analysis showed a strong correlation of IVIM parameters and biochemical indices of liver function, supporting its ability to evaluate liver functional recovery after PH.

Histologic analysis showed different regeneration modes between normal and fibrotic liver after left lateral lobe resection. In control group, LR was achieved solely by hepatocyte hypertrophy, which was consistent with previous study (Miyaoka et al., 2012). However, in fibrosis group, both hepatocyte hypertrophy and cell division were observed. On day 1 post-PH, hypertrophy of hepatocytes occurred in both normal and fibrosis groups, then recovered slowly. After day 3 post-PH, hepatocytes remained hypertrophic to some extent in fibrosis group, but continued to return back to baseline levels gradually in control group. In addition, hepatocyte diameter in the fibrosis group was significantly greater than that of controls at all time points post-PH. Hepatocyte hypertrophy is the respond to the immediate requirement to maintain homeostasis (Miyaoka et al., 2012). Liver function analysis in our animal study and previous clinical studies (Sugawara et al., 2002; Humar et al., 2005) showed a relatively low elevation and rapid recovery in normal liver after left lateral lobe liver resection. While, in fibrosis rats, liver function was damaged before surgery and further aggravated after surgery. Liver regeneration should be obvious to repair the damage and recover liver function. This might be the reason for greater hepatocyte hypertrophy in normal liver compared with fibrosis liver tissue. The increase of hepatocyte Ki-67 indices on day 2 post-PH in fibrosis group also indicated its higher LR activity.

This study had limitations. First, to achieve dynamic data of the MR parameters over continuous time points, the imaged animals did not undergo histologic analysis. Second, to reduce the number of animals used, a smaller number of rats were used at each time point for histologic analyses. Third, to increase survival rates in the fibrosis group, only the left lateral lobe was removed to establish a PH model. Further studies on varying levels of liver resection in fibrosis liver models are necessary.

## Conclusion

This study assessed the usefulness of IVIM imaging for the noninvasive assessment of liver regeneration and liver functional recovery after PH in control and fibrotic livers. After left lateral lobe resection, fibrotic livers regenerated by increasing hepatocyte size and cell division, but control liver regenerated only by increasing hepatocyte size. Liver D values increased, and PF values decreased immediately post-PH, then gradually returned to baseline levels. Liver PF values were higher in fibrotic livers compared with control livers post-PH. IVIM-derived MR parameters correlated well with LR-related histology and liver function biochemical results. IVIM imaging is a noninvasive and reliable method for monitoring the process of LR and functional recovery in control and fibrotic livers after PH.

## Data Availability Statement

The original contributions presented in the study are included in the article/Supplementary Material, further inquiries can be directed to the corresponding author.

## Ethics Statement

The animal study was reviewed and approved by the Ethics Committee of Tianjin First Central Hospital.

## Author Contributions

SX designed the study, carried out the experiments, analyzed the data, and drafted the manuscript. CQ carried out experiments and analyzed the data. YS, YY, KZ, LC, and YC carried out the experiments. ZH supported the pathological analysis. MB supported the animal surgery and pathological analysis. QZ supported the animal surgery. JZ and RG contributed with MRI protocol and revised the manuscript. WS designed the study and revised the manuscript. All authors contributed to the article and approved the submitted version.

## Conflict of Interest

JZ was employed by Siemens Healthcare (China), Beijing, China. RG was employed by Siemens Healthcare, Erlangen, Germany. The remaining authors declare that the research was conducted in the absence of any commercial or financial relationships that could be construed as a potential conflict of interest.

## Publisher’s Note

All claims expressed in this article are solely those of the authors and do not necessarily represent those of their affiliated organizations, or those of the publisher, the editors and the reviewers. Any product that may be evaluated in this article, or claim that may be made by its manufacturer, is not guaranteed or endorsed by the publisher.
