# Supplementary material for: Assessment of Fibrotic Liver Regeneration After Partial Hepatectomy With Intravoxel Incoherent Motion Diffusion-Weighted Imaging: An Experimental Study in a Rat Model With Carbon Tetrachloride Induced Liver Injury
Source: Front Physiol. 2022 Feb 18;13:822763. doi: 10.3389/fphys.2022.822763 (PMC8894856; doi:10.3389/fphys.2022.822763)
Supplement: Supplementary file 1 [file Data_Sheet_1.docx]

**Appendix**

**Table S1.** Intra-reader Agreement for Measurement of Liver Magnetic Resonance parameters

|  | ICC | | Bland-Altman | |
| --- | --- | --- | --- | --- |
| Parameter | ICC value | 95% CI | Mean of Difference | 95% agreement range |
| Fibrosis group |  |  |  |  |
| D | 0.861 | 0.601-0.951 | 0.020 | -0.133-0.173 |
| D^*^ | 0.891 | 0.687-0.962 | 0.580 | -22.25-23.41 |
| PF | 0.886 | 0.672-0.960 | 0.323 | -2.306-2.992 |
| Control group |  |  |  |  |
| D | 0.938 | 0.834-0.977 | 0.002 | -0.018-0.023 |
| D^*^ | 0.957 | 0.886-0.984 | -6.116 | -24.60-12.37 |
| PF | 0.947 | 0.859-0.980 | 0.281 | -2.581-3.142 |

**Table S2.** Inter-reader Agreement for Measurement of Liver Magnetic Resonance parameters

|  | ICC | | Bland-Altman | |
| --- | --- | --- | --- | --- |
| Parameter | ICC value | 95% CI | Mean of Difference | 95% agreement range |
| Fibrosis group |  |  |  |  |
| D | 0.905 | 0.750-0.966 | 0.015 | -0.085-0.115 |
| D^*^ | 0.842 | 0.547-0.945 | 0.024 | -27.04-27.09 |
| PF | 0.945 | 0.851-0.981 | 0.439 | -0.765-1.643 |
| Control group |  |  |  |  |
| D | 0.951 | 0.868-0.982 | -0.002 | -0.020-0.017 |
| D^*^ | 0.984 | 0.958-0.994 | 0.750 | -12.36-13.86 |
| PF | 0.984 | 0.958-0.994 | 0.136 | -1.512-1.785 |


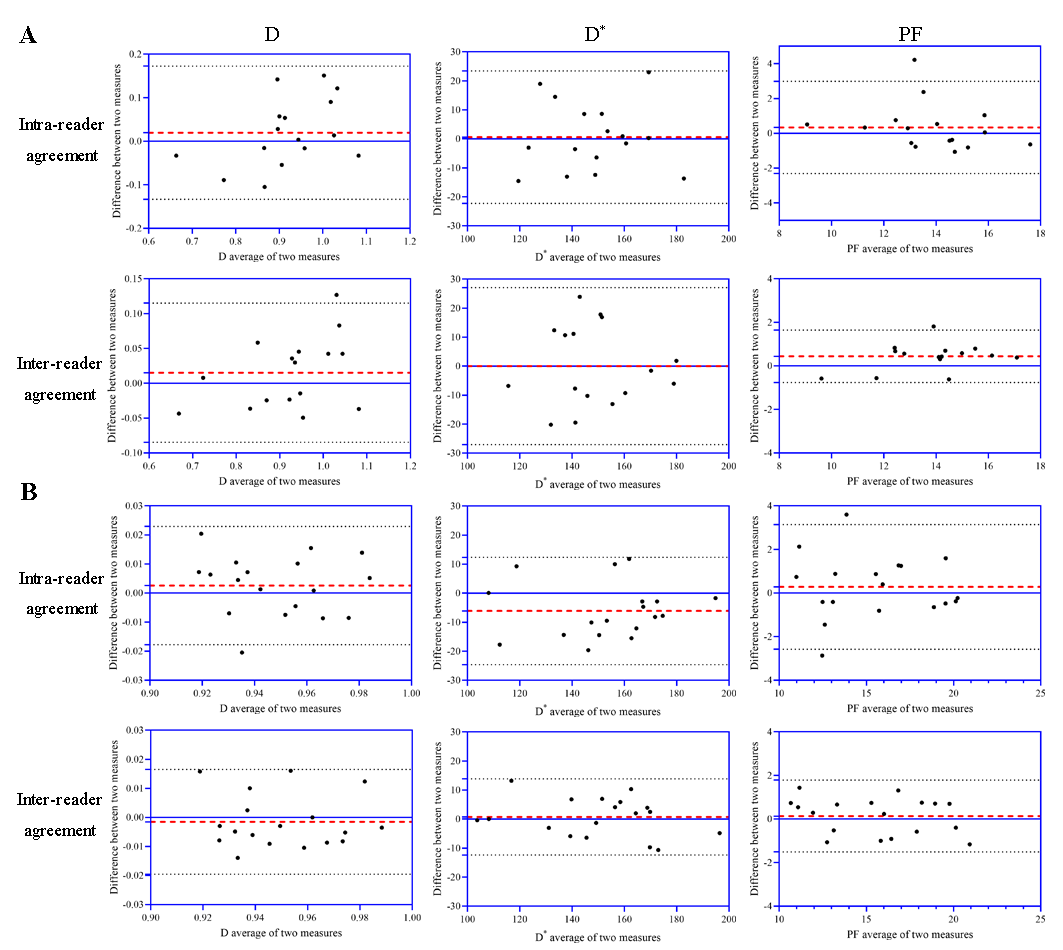
**Figure S1.** Bland-Altman plots for both readers in fibrosis rats (A) and control rats (B) . Upper row shows differences in the measurements of one reader for two measurements and lower row of two readers. Dotted lines represent 95% - confidence intervals
